# Weekly Training Load Differences between Starting and Non-Starting Soccer Players

**DOI:** 10.5114/jhk/171449

**Published:** 2023-11-28

**Authors:** Matej Varjan, Mikulas Hank, Maros Kalata, Paweł Chmura, Lucia Mala, Frantisek Zahalka

**Affiliations:** 1 Sport Research Centre, Faculty of Physical Education and Sport, Charles University, Prague, Czech Republic.; 2 Department of Team Games, Wroclaw University of Health and Sport Sciences, Wrocław, Poland.

**Keywords:** external load, monitoring, physical performance, player development

## Abstract

The aim of this study was to examine the differences in the weekly training load between starters and non-starters classified based on the match starting line-up, with respect to the playing position and a training day. Notably, 31 young adult soccer players (age: 18.79 ± 1.04 years) competing in the 3^rd^ Czech division were monitored across the season. The weekly training load was measured using a GPS system as follows: total distance covered (TD), high-speed running distance (HSR), sprint running distance (SR), and acceleration and deceleration distance (ACDC). We found higher values in three out of four observed variables (HSR, SR, and ACDC, excluding TD) for starters compared to non-starters (p < 0.05), with small to moderate effect sizes (d = 0.40–0.49). Differences were observed especially in players who were fullbacks, offensive midfielders, and forwards. Moreover, the largest differences were found in training prior to a match day for HSR, SR, and ACDC (p < 0.05). Non-starters experienced lower weekly external loads in offensive player positions, predominantly in high-intensity variables, which are essential for their physical performance. It seems that non-starters may experience potential under-loading in the training process. Coaches and practitioners should be aware of this potential risk and find an appropriate method to compensate for load discrepancies, particularly in terms of high-intensity activities.

## Introduction

Modern soccer requires significant emphasis on the daily monitoring of the players' load in training and not only during matches (Teixeira et al., 2021). It seems that substitutes and players who are not nominated in the starting line-up in a match (non-starters) experience significantly lower loads in weekly training micro-cycles when compared to nominated players (starters) (Casamichana et al., 2022; Stevens et al., 2017). Differences are observed mainly in the distance covered at higher speeds and the number of accelerations and decelerations (Dalen and Lorås, 2019; Nobari et al., 2020). Moreover, non-starters tend to be slower and have lower maximal aerobic capacity (Manson et al., 2014; Radzimiński et al., 2021), as well as poorer vertical jump performance (Magrini et al., 2018). Regardless of their “starting” status, optimal loading of all players in the training process can play a crucial role in enhancing physical performance and maintaining the health of players, especially when dealing with young adult players at the beginning of their professional careers (Gabbett, 2016).

Evolution in soccer over the last few years has shown that high-intensity activities are becoming the basis of match physical performance, as their proportion has increased the most. In contrast, the total distance covered did not vary significantly (Bush et al., 2015; Lago-Peñas et al., 2022). To monitor players’ loads and activity in a training process, GPS technology has become a traditional approach, which allows to record a wide range of external load variables. The most relevant and, according to Akenhead and Nassis (2016), the most widely used external load variables are total distance covered, distance covered at high speeds (above 19.8 km/h and 25.2 km/h) as well as acceleration variables. Therefore, we also decided to investigate these variables in our study, for the greatest possible impact and applicability to practice. According to Dalen and Lorås (2019), starters reached higher results in external load variables, especially in high-speed running distances and the number of accelerations and sprints. Similarly, Sams et al. (2020) found higher internal loads (rating of perceived exertion; RPE) across the season in starters when compared to substitutes and non-playing players. If the match load is added to the training load, the total weekly load will probably increase even further. Subsequently, the differences are primarily determined by the amount of time spent on the match (Casamichana et al., 2022; Dolański et al., 2018). Therefore, it is recommended for non-starters to compensate for the insufficient load with compensatory training (Hills et al., 2020a). However, even this may not fully compensate for the match load, and from a long-term perspective, non-starters could be potentially under-loaded (Calderon-Pellegrino et al., 2022; Stevens et al., 2017). Moreover, the days in a weekly micro-cycle differ not only in technical/tactical components, but also with respect to the physical load, such as the distance in total and at high speeds as well as accelerations and decelerations (Akenhead et al., 2016; Martín-García et al., 2018). The different training doses in a traditional single-match in-season micro-cycle result from the recovery needs at the beginning of the micro-cycle, however, the lowest external load is observed in training preceding the match. The “training peak” to maintain or develop physical performance is usually placed in the middle of the micro-cycle (mostly 3–4 days before the match). The distribution of the training load varies depending not only on the seasonal period, but also on the number of matches in a micro-cycle (Anderson et al., 2015; Malone et al., 2014). Therefore, when analysing intra-micro-cycle differences, it is necessary to consider training days (defined as a match day (MD) plus or minus) on which potential differences between starters and non-starters occur.

Optimisation of the training load with regard to the playing position is important because it significantly influences the physical load of players; additionally, it is recommended to consider the playing position when analysing performance of players in soccer (Marques et al., 2016). Previous studies have confirmed the differences in match and training loads with respect to the playing position; however, they did not consider the match starting status (Martín-García et al., 2018; Plakias, 2023; Vieira et al., 2019). This indicates a lack of studies considering the influence of the starting status on the weekly training load in soccer players. To the best of our knowledge, this is the first study providing comprehensive information on the training load of starters and non-starters while considering positional differences and training days. This complex information is essential for proper management of the training process.

Considering the above and the importance of an individual approach to players, the training load of starters and non-starters has become a relevant research-practice gap to control the training and match demands in soccer. Therefore, the aim of this study was to investigate the differences in the weekly training external load between starters and non-starters, with respect to the playing positions and training days in young adult soccer players. We assumed that significantly higher values of high-speed running distance, sprinting distance as well as acceleration and deceleration distance would be found in starters compared to non-starters.

## Methods

### Participants

The research sample consisted of 31 young adult male soccer players (age = 18.79 ± 1.04 years; body height = 182.13 ± 8.13 cm; body mass = 73.88 ± 7.13 kg) competing in the 3^rd^ Czech division. Participants belonged to the reserve team of a 1^st^ division team (participating in the UEFA European Cups). The analysed team won the league and was promoted to the 2^nd^ Czech division in the investigated 2021/2022 season. Moreover, nine players played for the Czech youth national teams (categories U18–U21) in that season. The inclusion criteria for each micro-cycle analysed were met by players who were registered and available for match play at the end of the micro-cycle. At the same time, only players who completed all five full training sessions in a given weekly micro-cycle were included in the analysis. All injured players, players in recovery, or players who had other health issues were excluded from the analysis of a given micro-cycle. Playing positions were determined on the basis of a stable 4-4-2 playing system as follows: central defender (CD, n = 5), full back (FB, n = 5), defensive midfielder (DM, n = 4), offensive midfielder (OM, n = 8), and forward (FW, n = 9) (Casamichana et al., 2022). The goalkeeper position was excluded from the analysis.

All participants and their legal representatives (for underage participants) were familiarised with the study. This study was approved by the ethics committee of the Faculty of Physical Education and Sport, Charles University in Prague, Czech Republic (approval number: 259/2020; approval date: 30 October 2020), and informed consent was obtained. This research was conducted in accordance with the ethical standards of the Declaration of Helsinki and research in sport science.

### Procedures

External load data were collected in all training sessions throughout the official 2021/2022 season. The data consisted of all the “on-pitch” training sessions in a particular micro-cycle. The match load data as well as the training load data from the strength development training sessions were not quantified and analysed in this study. Only training micro-cycles that had the structure presented in Table 1, with five soccer specific “on-pitch” training sessions, were analysed. The training load data were categorised with respect to the number of days after or before the match (MD plus or minus) (Akenhead et al., 2016).

**Table 1:** Structure of the habitual weekly micro-cycle (on-pitch training sessions), with mean values (mean ± SD) of external load variables and training duration.

	MD+1	MD−5	MD−4	MD−3	MD−2	MD−1
Training content	Day off	Recovery and skill; Passing drills; Positioning games	SSG (3v3, 4v4); Sprint, COD and plyometrics	MSG or LSG; Shooting,	Tactical focus – in game situations; Positioning games; Position-specific drills	Player cooperation - 11v11 from small to medium pitch sizes; Tactical focus – set pieces; Acceleration, reaction speed
Intensity		Low/Moderate	High	Moderate/high	Low	Moderate
Volume		Moderate	Moderate/high	High	Moderate	Low
Pitch size		Medium	Small	Medium/Large	Large	Small/Medium
Duration (min)		71.82 ± 18.11	82.32 ± 12.72	77.87 ± 19.26	85.71 ± 14.18	65. 52 ± 13.99
TD (m)		5098.54 ± 1009.95	5808.11 ± 1212.90	5977.29 ± 1414.09	4503.22 ± 1165.33	3649.54 ± 709.53
HSR (m)		144.83 ± 95.72	412.50 ± 186.00	337.46 ± 215.62	155.10 ± 114.82	63.71 ± 38.14
SR (m)		17.34 ± 22.19	92.37 ± 65.11	55.44 ± 55.44	28.20 ± 34.82	5.20 ± 8.33
ACDC (m)		111.43 ± 39.08	161.59 ± 40.93	122.86 ± 42.04	81.00 ± 40.30	68.58 ± 24.54

MD – match day; TD – Total distance; HSR – High speed running; SR – Speed running; ACDC – Acceleration and deceleration distance; SSG – Small-sided games; MSG – Medium-sided games; LSG – Large-sided games, COD – Change of direction

After accounting for the inclusion and exclusion criteria, we obtained 212 individual micro-cycle records (including the player's physical load during five full training sessions in a particular micro-cycle) during the study period (14 weeks). For each micro-cycle, participants were divided into two groups, starters and non-starters, according to the official league match that ended the weekly micro-cycle. Starters (120 individual micro-cycle records) consisted of players who were in the starting line-up. Non-starters (92 individual micro-cycle records) consisted of players who were substitutes or who were not nominated for the match (Manson et al., 2014). All players underwent the same training program, with no difference between starters and non-starters. Players who substituted or were not nominated for the match, were ensured to have undergone “top-up” training (not analysed in this study) to compensate for the limited match-play exposure immediately after the match or during the match day; therefore, it did not affect their training program in the following training micro-cycle. Compensatory training was designed to meet Martín-García's et al. (2018) recommendations to exceed 50% of match play values. It consisted of small-sided games and non-specific running exercises (duration = 40.13 ± 5.20 min, TD = 5832.09 ± 732.87 m, HSR = 621.75 ± 166.73 m, SR = 121.01 ± 37.49 m, ACDC = 173.52 ± 32.99 m). Players underwent all or part of this training, depending on the number of minutes played in the match.

### Measures

External load data were collected during the training sessions, which were performed on a natural grass surface within a pitch dimension of 105 × 68 m, according to the rules of the Football Association of the Czech Republic. Selected external load variables were obtained using 10-Hz GPS units integrated with a 400-Hz triaxial accelerometer (Playertek, Catapult, Australia). The 10-Hz GPS devices seemed to be the most valid and reliable for measuring the external load data in team sports (Callanan et al., 2021) and indicated a 2.5% estimation error in the distance covered, while the estimation error can increase up to 10.5% with increasing speed of movement (Rampinini et al., 2015). To avoid inter-unit errors, each player used the same GPS device throughout the research period. Players wore a specific fitted body vest with a bag placed on the upper back with the GPS unit (42 g, 84 × 42 × 21 mm) positioned inside. The GPS units were turned on 15 min prior to the match to ensure that the satellite connection was established. The external load data were downloaded retrospectively via the Playertek software for further analysis. We collected the following variables: total distance covered (TD), high speed running distance (HSR, > 19.8 km/h), sprint running distance (SR, > 25.2 km/h), and acceleration and deceleration distance (ACDC, > 3.0 m/s^2^) (Akenhead et al., 2016; Tierney et al., 2016).

### Statistical Analysis

Means with standard deviations were used for descriptive statistics. Verification of the normal data distribution was performed using the Shapiro-Wilk test. The two-sample *t*-test for independent groups (parametric data) or the Mann-Whitney U test (non-parametric data) was used to determine the statistical significance of differences in selected external load variables in the weekly micro-cycle between starters and non-starters. The Cohen’s *d* coefficient was applied to calculate the effect size and classified as large (*d*> 0.80), medium (0.50 <*d*< 0.80), small (0.20 <*d*< 0.50), or trivial (*d*< 0.20) (Cohen, 1992). Differences in the external load variables between training days were expressed using box plots. IBM SPSS® (IBM Statistical Package for Social Science® v. 25, Chicago, IL, USA) was used for all statistical analyses. To reject the null hypothesis, the *p*-value for all statistical analyses was set at *p*< 0.05.

## Results

Across the entire sample, we observed higher mean values for starters than for non- starters in TD (0.25%), HSR (14.00%), SR (25.17%), and ACDC (10.82%) (Table 2). There were statistically significant differences between groups for HSR, SR, and ACDC (*p*< 0.05), with small to moderate effect sizes (*d* = 0.40–0.49).

**Table 2:** Overall differences in weekly training load between Starters and Non-starters.

Variable	Mean ± SD		*p* value	Cohen’s *d*
	Starters (n = 120)	Non-starters (n = 92)		
TD	25063.74 ± 2607.84	25001.42 ± 2703.39	0.43	0.02
HSR	1176.32 ± 392.41	1031.83 ± 312.88	< 0.05	0.40
SR	217.52 ± 117.40	173.78 ± 87.42	< 0.05	0.41
ACDC	569.60 ± 126.41	513.99 ± 93.57	< 0.05	0.49

TD – Total distance covered; HSR – High-speed running distance; SR – Speed running distance; ACDC – Acceleration and deceleration distance

Table 3 reports differences in the weekly training load between starters and non-starters with respect to the playing positions. For CD and DM, we did not find differences between the groups in any of the examined variables (*p*< 0.05). FB of the starter group reached significantly higher TD and ACDC values compared to FB of the non-starter group (*p*< 0.05), with medium to large effect sizes (*d* = 0.60–0.92). For OM, we observed statistically significant differences in SR and ACDC (*p*< 0.05), with small to medium effect sizes (*d* = 0.39–0.57). FW of the starter group significantly outperformed FW of the non-starter group in HSR, SR and ACDC (*p*< 0.05), with medium to large effect sizes (*d* = 0.61–0.84).

**Table 3:** Differences in weekly training loads between Starters and Non-starters with respect to the playing positions.

Playing position	Variable	Mean ± SD		*p* value	Cohen's *d*
		Starters	Non-starters		
CD (n = 29)	TD	24092.43 ± 2081.03	24536.10 ± 2188.57	0.30	0.21
	HSR	973.25 ± 330.59	960.14 ± 286.66	0.46	0.04
	SR	172.40 ± 111.87	167.07 ± 96.39	0.45	0.05
	ACDC	505.40 ± 78.45	466.83 ± 103.61	0.13	0.44
FB (n = 44)	TD	26227.42 ± 2324.79	24608.06 ± 3301.95	< 0.05	0.60
	HSR	1352.22 ± 442.12	983.21 ± 329.93	< 0.05	0.91
	SR	264.91 ± 136.25	210.41 ± 107.11	0.09	0.43
	ACDC	615.01 ± 101.45	527.89 ± 80.11	< 0.05	0.92
DM (n = 35)	TD	25016.00 ± 2156.80	25563.08 ± 2455.69	0.25	0.24
	HSR	1000.79 ± 338.10	1025.25 ± 315.65	0.42	0.07
	SR	153.37 ± 102.74	154.19 ± 72.16	0.49	0.01
	ACDC	499.69 ± 97.89	521.66 ± 73.83	0.24	0.25
OM (n = 64)	TD	24411.09 ± 2691.13	24643.56 ± 2810.43	0.37	0.08
	HSR	1190.62 ± 352.94	1031.07 ± 318.96	< 0.05	0.48
	SR	212.13 ± 92.20	162.07 ± 84.95	< 0.05	0.57
	ACDC	572.46 ± 115.31	509.13 ± 92.86	< 0.05	0.39
FW (n = 40)	TD	25316.00 ± 3161.58	25919.60 ± 2285.70	0.25	0.21
	HSR	1272.16 ± 356.21	1126.77 ± 310.10	0.09	0.43
	SR	262.50 ± 102.05	183.50 ± 76.27	< 0.05	0.86
	ACDC	627.55 ± 169.60	532.32 ± 113.75	< 0.05	0.64

TD – Total distance; HSR – High-speed running distance; SR – Speed running distance; ACDC – Acceleration and deceleration distance; CD – Central defender; FB – Full back; DM – Defensive midfielder; OM – Offensive midfielder; FW – Forward. Bold font indicates significant difference

**Figure 1:**
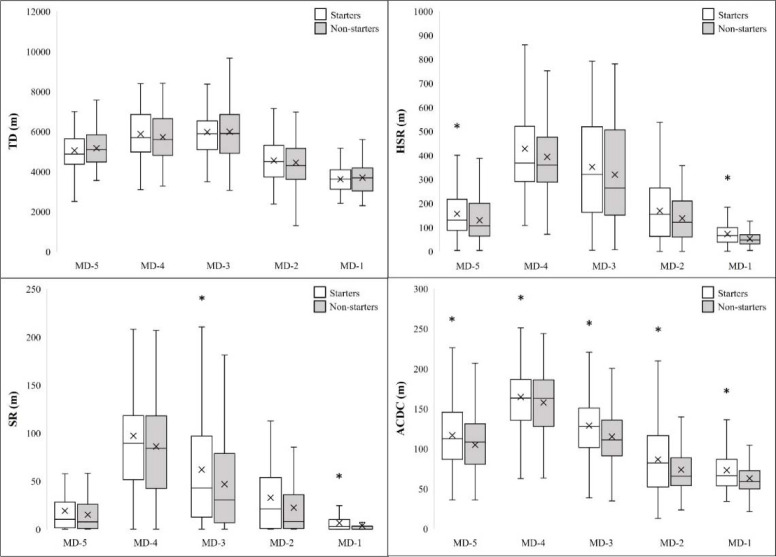
External load differences between Starters and Non-starters on different training days. TD – Total distance; HSR – High-speed running distance; SR – Speed running distance; ACDC – Acceleration and deceleration distance; CD – Central defender; FB – Full back; DM – Defensive midfielder; OM – Offensive midfielder; FW – Forward; * p < 0.05

A more detailed perspective on the differences between starters and non-starters when considering training days can be seen in Figure 1. Notably, excluding TD, where we did not observe differences between groups, starters outperformed non-starters on all training days, in HSR (9.21%–27.47%), SR (11.54%–45.02%), and ACDC (4.24%–14.76%), in terms of the mean values. For HSR, we observed significant between-group differences in MD−5 and MD−1 (*p*< 0.05). Regarding SR, there were significant differences in MD−3 and MD−1 (*p*< 0.05). Starters significantly outperformed non-starters in ACDC on all training days (*p*< 0.05).

## Discussion

This study examined the differences in the weekly training load between starters and non-starters across the full official season, with respect to the playing positions and a training day. Our main hypothesis was confirmed, as our results revealed significantly higher values in three out of the four observed external load variables (HSR, SR, and ACDC, excluding TD) for starters compared to non-starters (*p*< 0.05), and ACDC was found to be the most significant differentiating factor. Differences between the groups were observed especially in FB, OM, and FW players. Based on our findings, supported by previous studies (Alijanpour et al., 2022; Dalen and Lorås, 2019; Doyle et al., 2022; Nobari et al., 2020), it seems that non-starters may be systematically under-loaded in the training process, especially considering high-intensity variables, which are crucial for physical performance of players at offensive playing positions. Regardless of whether the reported lower physical fitness of non-starters is the cause or the consequence of systematically lower loading, it is essential to pay proper attention to this problem.

Different criteria were used to divide players into a starter and a non-starter group. If it is based only on the number of minutes played in a match, it may be unclear whether the player was the first choice of the coach and part of the starting line-up in a particular match (Casamichana et al., 2022; Dalen and Lorås, 2019; Sams et al., 2020; Stevens et al., 2017). To prevent this bias, we used a similar approach to classify players as starters or non-starters, as Magrini et al. (2018) or Manson et al. (2014) did.

When the match data were included in the weekly player load, starters reached higher loads, with the match time being the major dividing factor between starters and non-starters (Anderson et al., 2016; Casamichana et al., 2022; Stevens et al., 2017). Nevertheless, the differences between starters and non-starters can occur just in the training process, as reflected in our current findings and in previous research (Alijanpour et al., 2022; Dalen and Lorås, 2019; Doyle et al., 2022; Nobari et al., 2020). Even though all players followed the same training program between match days, we found significantly higher values for starters in HSR (14.00%), SR (25.17%), and ACDC (10.82%), with small to moderate effect sizes. We did not observe significant differences only in TD. Our findings are consistent with the weekly training results of Dalen and Lorås (2019), who reported lower total distance, high-speed running distance (*p*< 0.001, small effect size), and the number of accelerations and sprints (*p*< 0.001, small effect size) in non-starter junior soccer players. Similarly, non-starting professional players showed a lower number of accelerations and decelerations in the training process during early and mid-season (*p*< 0.05, moderate to very large effect sizes, except acceleration and deceleration over 4 m·s^−2^ during the mid-season) (Alijanpour et al., 2022) and over the entire season (*p*< 0.05, moderate to nearly perfect effect sizes) (Nobari et al., 2020), respectively.

Considering the playing positions in this research context makes our study unique. Higher values for starters were observed in FB, OM, and FW in contrast to CD and OM (*p*< 0.05), which corresponds with the offensive and defensive distribution of playing positions. A discussion may arise with FB as a primarily defensive position; however, in modern soccer (as well as in our case), FB usually replaces the position of the offensive wing player. Physical performance of offensive players is characterised by a higher amount of high-intensity movements (Bujnovsky et al., 2019), in which we found significantly higher values for starters at FB, OM, and FW positions (HSR: *d* = 0.43–0.91, SR: *d* = 0.43–0.86, and ACDC: *d* = 0.39–0.92). This means that differences between starters and non-starters were found in performance variables, which are crucial for these playing positions.

Looking at the structure of the micro-cycle and the distribution of training loads over the week, we observed an increased load in training on MD−4 and MD−3, especially in HSR, SR, and ACDC, and a subsequent decrease in loading as the match day approached. This trend is not surprising and has been previously observed (Stevens et al., 2017; Swallow et al., 2021). Notably, ACDC was the only variable in which non-starters recorded a lower load across all training days (*p*< 0.05). An increased number of high accelerations and decelerations can be a sign of increased effort and activity of the player. This activity may also play a role in the selection of players for the match starting line-up and could be considered a key performance indicator in soccer. Moreover, we observed the largest differences in MD−1 training, as non-starters reached significantly lower values in three out of the four observed variables (HSR, SR, and ACDC). MD−1 consists of training focused mostly on tactical preparation for the next match, but the objective was also to increase the player cooperation on the field (especially those who played together in the starting line-up) and let them feel the match mood. When playing 11 vs. 11 from small to medium sized pitches and practicing set-pieces, players who were not part of the starting line-up in the match filled the role of the opposing team and took over their tactical behaviour (based on the coach's instructions). This may be one of the reasons why on MD−1, players who are not in the starting line-up experienced a different, usually lower training load. In contrast to our findings, a study that examined only one isolated micro-cycle in international women soccer players did not observe differences in the external load between starters and non-starters on any training day (Doyle et al., 2022).

The most frequent strategies used by practitioners (senior and academy level) to compensate for insufficient loading in non-starters were compensatory training sessions, extra conditioning during the week or the use of small-sided games (Hills et al., 2020b). In this context, Casamichana et al. (2022) claim that MD−1, MD, and MD+1 sessions could be the most appropriate to try to compensate for the absence of competitive stimuli. Our study is consistent with this conclusion, as non-starters underwent compensatory training always on MD, and it usually consisted of the extensive type of small-sided games, due to achieving a higher external load compared to the intensive type, while maintaining a high internal load (Zanetti et al., 2022); compensatory training was supported by non-specific running exercises (not quantified in this study). Similarly, high-intensity interval training (HIIT) is also considered a suitable method of load supplementation (Buchheit, 2019). However, despite the efforts, it is still challenging to fully compensate for the insufficient match loads and this problem persists (Hills et al., 2020a). The match load and match experience are essential for players’ development, and it will be a future challenge to find appropriate ways to fully replace them.

The study limitations include small sporadic changes in the reported training structure that resulted from the actual needs of the team and particular players. For future research, it may be beneficial to consider short-term changes in players' playing positions when analysing training loads, although in our study these changes were minimal and did not affect the results of the research. Generalising the results of our study should be approached with caution, as the study is only reflective of one team; therefore, it is not necessarily representative of training requirements of other teams or categories. We strongly believe that it is relevant to demonstrate the existential risk of these differences. This study only analysed the specific training load, which we believe is important, but also considering the match load, compensatory training, and strength development training could provide a more comprehensive picture of the weekly load and differences between starters and non-starters. Finally, we only analysed a traditional weekly micro-cycle with five training sessions; however, there are other types of micro- cycles that need to be analysed. We investigated only inter-individual differences in player loads, but we suggest that future studies should also consider intra-individual differences within and between training micro-cycles. A challenge for future research will be to identify the reasons behind the occurrence of such differences between starters and non-starters in the training process, as the explanation remains unclear. There is also a need to find appropriate methods to fully compensate for the match load and thus, improve the training process and players’ development.

## Conclusions

Our findings highlight the importance of monitoring the external load of daily training for managing and optimising the soccer training process. Significant differences in the weekly training load between starters and non-starters were found. Non-starters experienced lower external loads and under-loading in offensive playing positions, predominantly in high-intensity variables (HSR, SR, and ACDC), which are essential for physical performance of these playing positions. Acceleration and deceleration distance was the strongest distinguishing variable between the groups. The largest differences between starters and non-starters were observed in MD−1. There is a risk of cumulative load differences between starters and non-starters, which may lead to systematic under-loading and poor physiological adaptations in the long term, especially in offensive playing positions. It is essential to assess player’s loads individually and position-specifically, but not only on a short-term basis, where differences may appear negligible, but also on a weekly, monthly or seasonal basis. In this context, the results of previous studies should be taken into account as when we include the match load in the weekly load, the differences between starters and non-starters may become even more significant. Coaches and practitioners should be aware of this potential risk and consider compensating for the insufficient load with additional physical training activities (preferably under soccer-specific conditions and in high-intensity activities), and by arranging training matches or allowing players to play for lower teams or categories.
